# First-Principles Guided Design of Pd-Decorated V_2_O_5_/Porous Silicon Composites for High-Performance NO_2_ Sensing at Room Temperature

**DOI:** 10.3390/nano15070513

**Published:** 2025-03-28

**Authors:** Xiaoyong Qiang, Yongliang Guo, Zhipeng Wang, Tao Chen, Rui Zhang, Weibin Zhou

**Affiliations:** College of Electronic Information and Automation, Tianjin University of Science and Technology, Tianjin 300457, China; shawn@tust.edu.cn (X.Q.); chentao@tust.edu.cn (T.C.); zhangrui@tust.edu.cn (R.Z.)

**Keywords:** NO_2_ gas sensor, Pd decoration, V_2_O_5_/PSi composite, first-principles calculations, room-temperature sensing

## Abstract

A Pd-decorated V_2_O_5_/porous silicon (Pd-V_2_O_5_/PSi) composite was synthesized via magnetron sputtering for enhanced NO_2_ gas sensing. The material’s morphology and composition were systematically characterized, and its gas sensing performance was evaluated through comprehensive experimental measurements and first-principles calculations. The decoration of Pd nanoparticles significantly improved the sensing capabilities of the V_2_O_5_/PSi composite, particularly enhancing sensitivity and response/recovery characteristics. Experimental results revealed a 3.1-fold increase in response to specific NO_2_ concentrations (ppm level) compared to the undecorated V_2_O_5_/PSi sensor. The composite exhibited rapid NO_2_ response at room temperature with excellent selectivity, reproducibility, and long-term stability. First-principles calculations elucidated the structural, electronic, and adsorption properties of the Pd-V_2_O_5_/PSi composite, uncovering the gas sensing mechanism in NO_2_ environments. This combined experimental and theoretical study provides valuable insights for developing advanced gas sensors and lays a foundation for optimizing metal oxide-based sensing materials.

## 1. Introduction

The rapid advancement of industrialization has exacerbated environmental pollution, necessitating the development of efficient technologies for real-time monitoring of toxic gases. Nitrogen oxides (NOx), particularly nitrogen dioxide (NO_2_), are among the most concerning atmospheric pollutants due to their high toxicity, chemical stability, and persistence in the environment. These characteristics pose significant risks to both environmental health and human well-being, driving the urgent need for sensitive, stable, and efficient NO_2_ gas sensors. Metal oxide semiconductors (MOS), such as WO_3_, ZnO, TiO_2_, SnO_2_, CuO, and V_2_O_5_, have emerged as pivotal materials in gas sensor technology, owing to their excellent electrical properties, high gas sensitivity, compatibility with fabrication processes, and promising gas sensing capabilities [1,2,3,4,5,6,7]. However, despite their advantages, these materials often face challenges such as limited sensitivity at low concentrations or poor selectivity in complex gas mixtures. Among these, V_2_O_5_, an n-type semiconductor, has garnered significant attention due to its superior catalytic activity, favorable electrical properties, and versatile surface chemistry, making it a particularly promising candidate for NO_2_ detection. Nevertheless, the practical application of pure V_2_O_5_ in gas sensing is often hindered by its moderate responsiveness and selectivity, especially for NO_2_ detection, underscoring the need for further material optimization.

To address these limitations, researchers have explored various strategies to enhance the gas-sensing properties of MOS materials, including morphological engineering, heterostructure construction, and noble metal decoration [8,9,10,11,12,13,14]. Among these strategies, noble metal decoration has proven particularly effective in enhancing gas-sensing performance. This approach introduces additional active sites, facilitates gas molecule adsorption and reaction, and improves sensing capabilities through catalytic and spillover effects [15,16,17,18,19]. Notably, Pd decoration has demonstrated exceptional promise [20,21,22,23,24]. For instance, Wu et al. reported that Pd/PdO-functionalized NiFe_2_O_4_ nanoparticles synthesized via a nonaqueous method exhibit excellent n-butanol sensing performance, including high response, fast response/recovery times, and superior selectivity, attributed to catalytic effects and heterojunction formation [20]. Wang et al. demonstrated that Pd-doped GeSe monolayers exhibit enhanced adsorption and sensing properties for greenhouse gases (NO_2_ and SF_6_), making them promising candidates for low-power resistive gas sensors and gas purification applications [23]. Hung et al. reported that Pd-NiO nanorods/SnO_2_ nanowires sensors exhibit a 3.72-fold and 6.53-fold enhancement in acetone response compared to pristine SnO_2_ and NiO, respectively, with a response of 14.88 to 500 ppm acetone and fast response/recovery times of 11/150 s [24].

Porous silicon (PSi) has emerged as a promising substrate for gas sensing applications due to its unique structural properties, including a high specific surface area, tunable pore dimensions, and excellent chemical stability [25,26,27]. These characteristics enable PSi to provide abundant gas adsorption sites and optimized gas diffusion pathways, making it an ideal platform for integrating with metal oxides to form composite sensing materials. Recent studies have demonstrated the enhanced performance of PSi-based composites [25,28]. For example, Gonzalez et al. showed that Pt-catalyzed SnO_2_/porous silicon hybrid structures exhibit a 7.5-fold increase in CO_2_ sensitivity at 100 °C, attributed to enhanced charge carrier exchange and high surface area provided by porous silicon [25]. Almansba et al. demonstrated that CuO/Co_3_O_4_/PSi thin films exhibit remarkable NH_3_ sensing performance at room temperature, achieving a 200% response to 170 ppm NH_3_ with ultrafast response/recovery times, highlighting the potential of nanocomposite materials for ammonia detection [28]. These composites leverage the synergistic effects of PSi’s porous architecture and the catalytic properties of metal oxides, resulting in improved sensitivity, selectivity, and response time.

In this study, we present a first-principles-guided design of Pd-V_2_O_5_/PSi composites for high-performance NO_2_ sensing at room temperature. The composite was fabricated using magnetron sputtering, and its structural, morphological, and electronic properties were systematically characterized. Comprehensive experimental measurements demonstrated that Pd decoration significantly enhances the sensitivity, selectivity, and response/recovery characteristics of the V_2_O_5_/PSi composite. First-principles calculations were employed to elucidate the underlying mechanisms, revealing the structural, electronic, and adsorption properties of the Pd-V_2_O_5_/PSi system. These theoretical insights, combined with experimental results, provide a detailed understanding of the interaction between NO_2_ molecules and the composite surface, highlighting the role of Pd in improving gas adsorption and electron transfer. By integrating experimental and theoretical approaches, this study establishes a robust framework for understanding and enhancing gas sensing mechanisms, contributing to the broader field of gas sensor technology and paving the way for practical applications in environmental monitoring and public health protection.

## 2. Materials and Methods

### 2.1. Preparation of Pd-V_2_O_5_/PSi Composite

**Figure 1:**
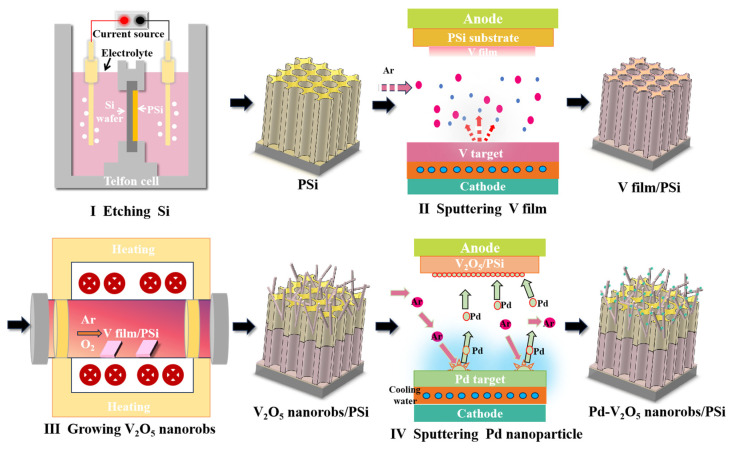
Schematic illustration of the preparation process for Pd-V_2_O_5_/PSi.

The materials and reagents used in this study were rigorously selected to ensure high purity and effectiveness. PSi substrates were fabricated via electrochemical etching using a p-type silicon wafer (resistivity: 10–15 Ω·cm, thickness: 400 ± 10 μm, purity: 99.9999%) in an electrolyte solution of hydrofluoric acid (HF, 48 wt.%) and N,N-Dimethylformamide (DMF, 99.5 wt.%) at a volume ratio of 1:3. A constant current density of 110 mA/cm^2^ was applied for 8 min, resulting in a uniform porous structure with an average pore diameter of 75 ± 15 nm and a porosity of 50–60%. After etching, the PSi substrates were rinsed with deionized water and dried under nitrogen flow to ensure a clean surface for subsequent depositions. Next, V thin films were deposited onto the PSi substrates using a DC magnetron sputtering system. The chamber was evacuated to a background pressure below 1 × 10^−4^ Pa, and 50 sccm high-purity Ar (99.999%) was introduced to maintain a working pressure of 2 Pa. A V target (99.99% purity) was used, and the deposition was performed with a DC power of 100 W, achieving a deposition rate of approximately 0.5 nm/s to form a 100 nm V film. Subsequently, the PSi/V samples were thermally annealed in a tube furnace at 550 °C for 30 min in a mixed atmosphere of O_2_ (20%) and Ar (80%) with a total gas flow rate of 100 sccm, converting the V films into V_2_O_5_ nanorobs. The heating and cooling rates were set to 5 °C/min to minimize thermal stress and ensure uniform oxidation. Finally, Pd nanoparticles were decorated onto the PSi/V_2_O_5_ samples using the same magnetron sputtering system. The chamber was evacuated to a background pressure below 1 × 10^−4^ Pa, and Ar (99.999%) was introduced to achieve a working pressure of 0.5 Pa. A Pd target (99.99% purity) was used, and the deposition was performed with a DC power of 110 W for 10 s, ensuring uniform decoration of Pd nanoparticles without agglomeration. The detailed preparation process of the Pd-V_2_O_5_/PSi composite is illustrated in Figure 1, highlighting the key steps of PSi substrate fabrication, V thin film deposition, thermal annealing, and Pd nanoparticle decoration.

### 2.2. Fabrication of Gas Sensor Devices and Gas-Sensing Measurements

**Figure 2:**
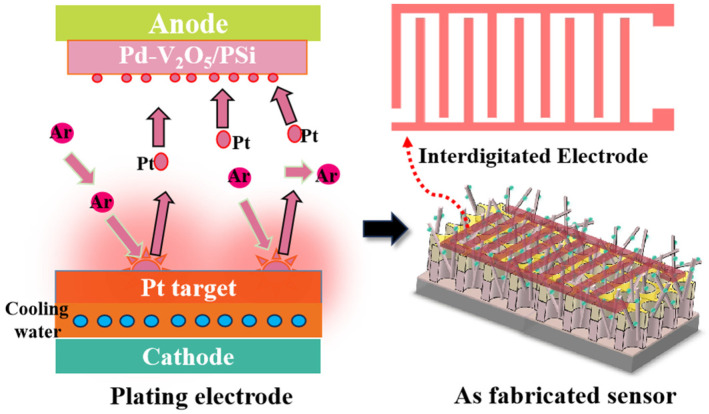
Schematic illustration of the Pd-V_2_O_5_/PSi sensor fabrication process.

Gas sensor devices were fabricated using the Pd-V_2_O_5_/PSi composite. As illustrated in Figure 2, interdigitated electrodes were deposited on the composite surface by DC magnetron sputtering with a Au target (99.99% purity). The electrode pattern, featuring a finger width of 250 μm and spacing of 50 μm, was defined using standard photolithography. The fabricated sensor was mounted on a custom-built gas sensor testing system for performance evaluation.

**Figure 3:**
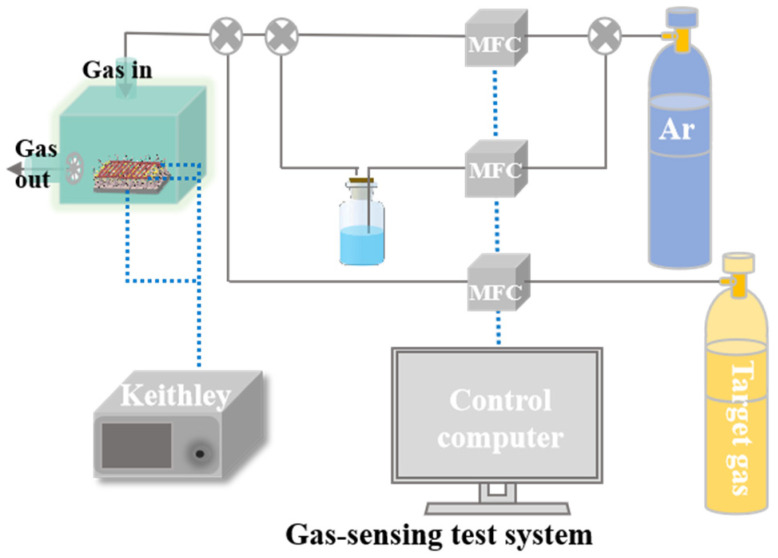
Schematic illustration of gas-sensing test system.

The gas sensing performance of the Pd-V_2_O_5_/PSi composite was evaluated using a custom-built testing system, as shown in Figure 3. The system comprises a temperature-controlled chamber and a precision data acquisition unit, enabling precise control of gas concentration (0.1–200 ppm) and operating temperature (0–300 °C). During testing, target gases were introduced into the chamber via mass flow controllers, and the sensor response was calculated as R_a_/R_g_, where R_a_ and R_g_ denote the sensor resistance in air and target gas, respectively. The response time was defined as the time required to reach 90% of the final equilibrium value, while the recovery time was defined as the time needed to return to 10% above the baseline resistance. All measurements were conducted at a relative humidity of 40 ± 5%, and each data point represents the average of at least three independent measurements to ensure reproducibility.

### 2.3. First-Principles Calculations

First-principles calculations were performed using the Vienna Ab Initio Simulation Package (VASP) within Materials Studio, employing the Projector Augmented-Wave (PAW) method to simulate electron–ion interactions. The exchange-correlation potential was described using the Perdew-Burke-Ernzerhof (PBE) functional within the Generalized Gradient Approximation (GGA), with van der Waals (vdW) interactions incorporated via the DFT-D3 method to ensure accurate modeling of gas adsorption phenomena. A plane-wave energy cutoff of 571.40 eV was applied to ensure convergence of total energy and forces. The Brillouin zone was sampled using a Γ-centered k-point grid, with a 1 × 1 × 1 k-point mesh used for both geometry optimization and electronic structure calculations. Geometry optimization was carried out with a convergence criterion of 1 × 10^−5^ eV/atom, and a vacuum layer of 20 Å was introduced along the z-axis to minimize periodic image interactions [29].

## 3. Results and Discussion

### 3.1. Structural and Morphological Characterization

**Figure 4:**
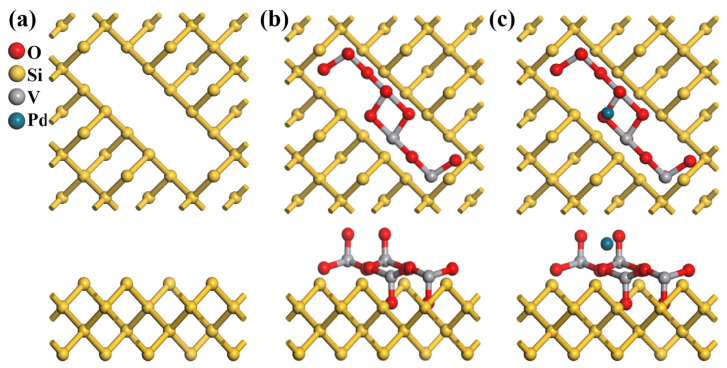
Atomic model (top view and side view) of (**a**) PSi, (**b**) V_2_O_5_/PSi, and (**c**) Pd-V_2_O_5_/PSi.

**Figure 5:**
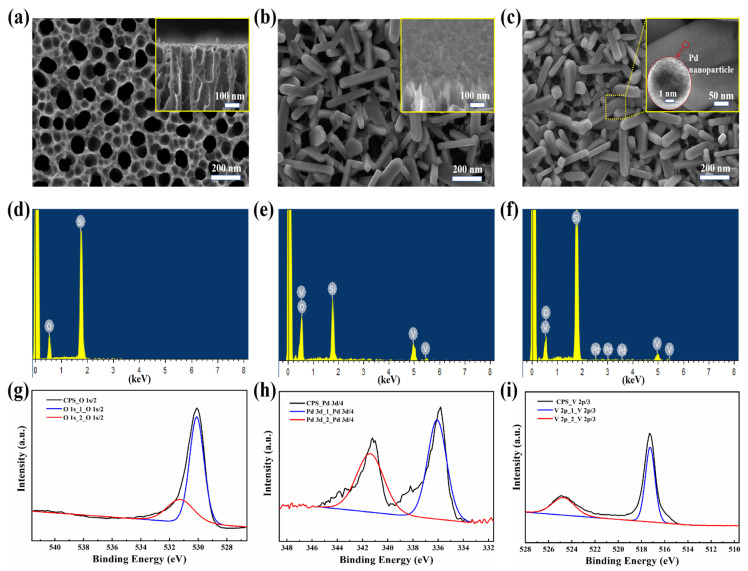
SEM images and EDS maps of (**a**,**d**) PSi, (**b**,**e**) V_2_O_5_/PSi and (**c**,**f**) Pd-V_2_O_5_/PSi; XPS spectrum of Pd-V_2_O_5_/PSi: (**g**) O 1s spectrum, (**h**) Pd 3d spectrum, (**i**) V 2p spectrum.

The atomic models in Figure 4a–c depict the structural evolution from pristine PSi to PSi/V_2_O_5_ and Pd-PSi/V_2_O_5_. To construct the PSi framework (Figure 4a), we initiated the simulation with a bulk crystalline silicon structure and introduced controlled pore architecture, with pore dimensions calibrated against experimental data. The simulated pore diameter represents a single pore for computational tractability, whereas experimental SEM (Figure 5a) shows an average pore size of 75 ± 15 nm across a macroporous matrix, as confirmed by statistical analysis of 50 pores via ImageJ 1.53. In the PSi/V_2_O_5_ structure (Figure 4b), the V_2_O_5_ framework retains its original arrangement, whereas in the Pd-decorated structure (Figure 4c), a Pd atom is embedded within theV_2_O_5_ layer, causing localized distortions. From the top view, the Pd atom disrupts the coordination of adjacent oxygen atoms. The side view further emphasizes this interaction, revealing the Pd atom forming bonds with oxygen atoms within the V_2_O_5_ network. This structural modification suggests that Pd decoration alters the electronic properties and enhances the catalytic performance of the material.

As shown in Figure 5a, the SEM image of the bare PSi substrate demonstrated smooth pore walls with uniform spatial distribution, exhibiting an average pore diameter of 75 ± 15 nm. The higher-magnification inset image further confirmed the vertically aligned pore channels, consistent with the anisotropic etching mechanism described in Section 2.1. SEM observations in Figure 5b revealed that the V_2_O_5_ nanorobs formed a homogeneous and continuous coating on the PSi substrate surface at specific angles, with the majority of V_2_O_5_ nanorobs preferentially growing on the PSi surface rather than within the holes. The inset of Figure 5b provides a cross-sectional view, further illustrating the uniform distribution of V_2_O_5_ nanorobs, which exhibit a width of 50–100 nm and a length of 200–400 nm. This configuration significantly increased the contact area between NO_2_ molecules and both the V_2_O_5_ nanorobs and PSi. As shown in Figure 5c, subsequent decoration with Pd nanoparticles via magnetron sputtering yielded uniform dispersion across the V_2_O_5_ nanorobs surface without observable aggregation, with Pd nanoparticles having an average diameter of approximately 5 nm (inset of Figure 5c). This resulted in a more compact and uniform surface morphology, enhancing the material’s gas sensing performance. Figure 5d–f presents the EDS spectra of PSi, PSi/V_2_O_5_, and Pd-PSi/V_2_O_5_. The Si element originates from PSi, the O and V elements are derived from the V_2_O_5_ nanorobs, and the Pd element comes from the Pd nanoparticles.

To further confirm the chemical composition and oxidation states of the Pd-V_2_O_5_/PSi composite, X-ray photoelectron spectroscopy (XPS) analysis was performed, and the results are presented in Figure 5g–i. The XPS spectra reveal distinct peaks corresponding to O 1s, Pd 3d, and V 2p core levels. The O 1s spectrum shows two primary peaks at 530.1 eV (lattice oxygen, O^2−^) and 531.3 eV (surface-adsorbed oxygen species, O^−^), confirming the presence of reactive oxygen species critical for gas sensing. The Pd 3d spectrum exhibits peaks at 336.1 eV (Pd 3d_5/2_) and 341.4 eV (Pd 3d_3/2_), corresponding to metallic Pd^0^. The V 2p spectrum shows peaks at 517.2 eV (V 2p_3/2_) and 524.5 eV (V 2p_1/2_), corresponding to the V^5+^ oxidation state in V_2_O_5_, confirming the phase purity of the material. The combination of SEM, EDS, and XPS analyses provides comprehensive evidence for the structural, morphological, and chemical properties of the Pd-V_2_O_5_/PSi composite, supporting its enhanced gas sensing performance.

### 3.2. Electronic Properties and Band Structure Analysis

**Figure 6:**
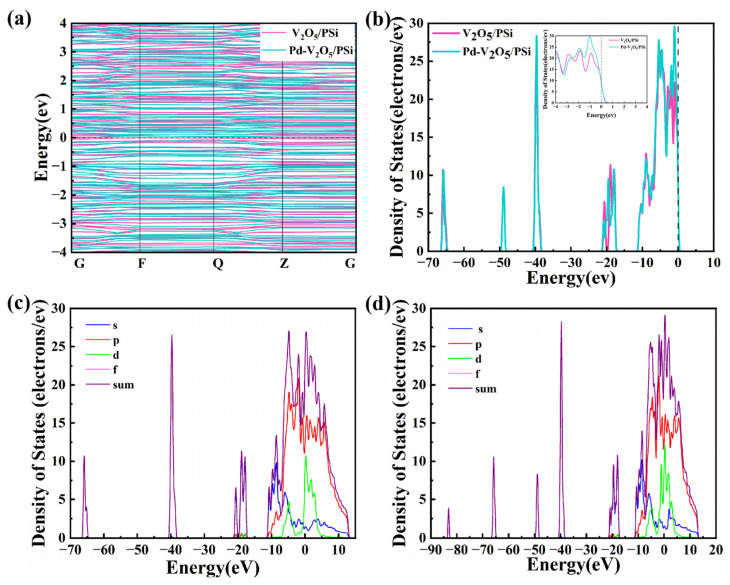
Electronic structure analysis of V_2_O_5_/PSi and PdV_2_O_5_/PSi composites: (**a**) Energy band structure and (**b**) DOS of V_2_O_5_/PSi and Pd-V_2_O_5_/PSi; PDOS of (**c**) V_2_O_5_/PSi and (**d**) Pd-V_2_O_5_/PSi.

Figure 6a,b present the energy band structure and density of states (DOS) of V_2_O_5_/PSi before and after Pd decoration, respectively. In Figure 6a, the band structure of V_2_O_5_/PSi exhibits a distinct bandgap, confirming its typical semiconducting behavior. The clear separation between the conduction band (CB) and valence band (VB) highlights limiting electronic transitions and resulting in relatively low conductivity. In contrast, Pd-V_2_O_5_/PSi demonstrates a reduced bandgap and the introduction of localized electronic states near the CB and within the bandgap. The DOS plot in Figure 6b shows a notable increase in states near the Fermi level, attributed to the decoration of Pd, thereby enhancing electron transport properties, making the Pd-V_2_O_5_/PSi system more suitable for applications such as catalysis and gas sensing. The projected density of states (PDOS) analysis in Figure 6c,d reveal distinct orbital contributions between the V_2_O_5_/PSi system (Figure 6c) and Pd-V_2_O_5_/PSi system (Figure 6d). In the V_2_O_5_/PSi system, the p-orbital exhibits hybridization near the Fermi level (0 eV), with contribution extending from −20 to +15 eV. The d-orbital of V_2_O_5_ contributes primarily between −20 and −10 eV, modulating interfacial charge transfer. Upon Pd decorating (Figure 6d), the p-orbital shows intensified hybridization near the Fermi level, indicating strengthened O–Si–V_2_O_5_ interactions. Most notably, the d-orbital density surges at 0 eV, attributed to Pd d-states, which directly facilitates electron transfer at the Pd-V_2_O_5_ interface. These coordinated enhancements in s/p/d-orbital densities, particularly the Pd-induced d-state prominence near the conduction band, collectively optimize charge transport kinetics and catalytic activity.

### 3.3. Gas-Sensing Performance

**Figure 7:**
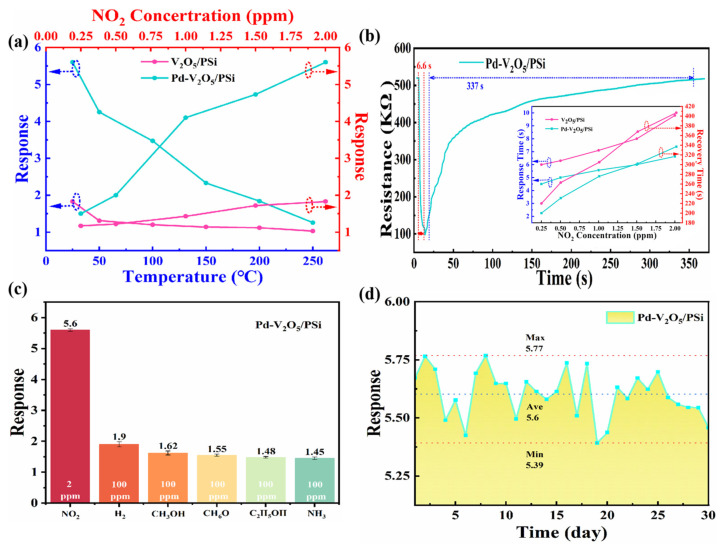
Gas sensing performance of V_2_O_5_/PSi and Pd-V_2_O_5_/PSi composites: (**a**) temperature and concentration dependence, including the response of V_2_O_5_/PSi and Pd-V_2_O_5_/PSi at various temperatures (25–250 °C) and to different V_2_O_5_ concentrations (0.25–2 ppm) at 25 °C; (**b**) dynamic response curve of Pd-V_2_O_5_/PSi to 2 ppm NO_2_ at 25 °C (inset: Response and recovery times for V_2_O_5_/PSi and Pd-V_2_O_5_/PSi to NO_2_ concentration (0.25–2 ppm) at 25 °C); (**c**) selectivity of Pd-V_2_O_5_/PSi to various gases at 25 °C; (**d**) long-term stability of Pd-V_2_O_5_/PSi to 2 ppm NO_2_ at 25 °C over 30 days.

Figure 7 presents a comprehensive evaluation of the sensing performance of V_2_O_5_/PSi and Pd-V_2_O_5_/PSi composites. As shown in Figure 7a, the temperature-dependent response characteristics of both composites were systematically investigated. The temperature sweep was performed at a fixed NO_2_ concentration of 2 ppm to ensure unambiguous interpretation of thermal effects. The sensor responses of both V_2_O_5_/PSi and Pd-V_2_O_5_/PSi exhibited a decreasing trend with increasing temperature from 25 °C to 250 °C, suggesting that room temperature (25 °C) represents the optimal operating condition for these sensors. This behavior can be attributed to several factors: (1) at higher temperatures, the thermal energy of NO_2_ molecules increases, leading to faster desorption from the sensor surface and reducing the extent of surface reactions; (2) the intrinsic carrier concentration in V_2_O_5_ increases with temperature, elevating the baseline conductivity and diminishing the relative resistance change; (3) the adsorption of oxygen species becomes less favorable at elevated temperatures, reducing the availability of pre-adsorbed oxygen ions critical for surface reactions; and (4) the catalytic efficiency of Pd nanoparticles may decrease at higher temperatures due to partial sintering or agglomeration. At this optimized temperature (25 °C), the concentration-dependent response was further examined across a range of NO_2_ concentrations (0.25–2 ppm). Both composites demonstrated increasing response signals with elevated NO_2_ concentrations. Specifically, the V_2_O_5_/PSi composite exhibited responses of 1.17, 1.22, 1.43, 1.72, and 1.83 for NO_2_ concentrations of 0.25, 0.5, 1, 1.5, and 2 ppm, respectively. In contrast, the Pd-V_2_O_5_/PSi composite showed significantly enhanced responses of 1.5, 2, 4.1, 4.73, and 5.6 for the same NO_2_ concentrations. Notably, at 2 ppm NO_2_, the response of Pd-V_2_O_5_/PSi reached 5.6, which is 3.1 times higher than that of the undecorated V_2_O_5_/PSi (1.83). This substantial improvement in sensitivity, particularly at lower concentrations, highlights the effectiveness of Pd decoration in enhancing the sensing performance of the composite material.

The dynamic response characteristics, particularly response and recovery times, were thoroughly investigated as shown in Figure 7b. From the dynamic response curve of Pd-V_2_O_5_/PSi to 2 ppm NO_2_ at 25 °C, it can be observed that the resistance decreases upon exposure to NO_2_, and after the response and recovery process, the resistance returns to its initial value. This behavior confirms the reversible nature of the sensing mechanism and the stability of the Pd-V_2_O_5_/PSi composite. The original response and recovery times for V_2_O_5_/PSi and Pd-V_2_O_5_/PSi to NO_2_ concentrations (0.25–2 ppm) at 25 °C are included as an inset. The Pd-V_2_O_5_/PSi composite demonstrated superior performance, exhibiting both faster response rates and shorter recovery times compared to the V_2_O_5_/PSi. Specifically, the response/recovery times of Pd-V_2_O_5_/PSi for NO_2_ concentrations of 0.25, 0.5, 1, 1.5, and 2 ppm were 4.5/200 s, 5.0/231 s, 5.5/276 s, 6.0/302 s, and 6.6/337 s, respectively. In contrast, the V_2_O_5_/PSi composite exhibited longer response/recovery times of 6.0/220 s, 6.3/263 s, 7.1/305 s, 8.0/368 s, and 9.8/407 s for the same NO_2_ concentrations. This significant improvement in response and recovery kinetics highlights the enhanced performance of the Pd-decorated composite. The enhancement can be attributed to the catalytic effect of Pd nanoparticles, which facilitate the adsorption and surface reaction kinetics of NO_2_ molecules on the V_2_O_5_ surface, leading to faster and more efficient gas sensing. Additionally, the porous structure of PSi provides abundant adsorption sites and optimized gas diffusion pathways, while the Pd-V_2_O_5_ heterojunction promotes efficient charge transfer and stabilizes the hole accumulation layer, further enhancing the sensing performance. In Table 1, we can see that in low concentration detection, Pd-V_2_O_5_/PSi exhibited better gas sensing performance than most of the previously reported composites.

Selectivity, a crucial parameter for practical sensor applications, was systematically evaluated as presented in Figure 7c. The gas sensing performance was tested against various interfering gases (H_2_, CH_3_OH, C_3_H_6_O, C_2_H_5_OH, NH_3_) at a concentration of 100 ppm, significantly higher than the target NO_2_ concentration (2 ppm). The results revealed that the Pd-V_2_O_5_/PSi composite maintained excellent selectivity towards NO_2_, with response values of 1.9, 1.62, 1.55, 1.48, and 1.45 for H_2_, CH_3_OH, C_3_H_6_O, C_2_H_5_OH, and NH_3_, respectively. In contrast, the response to 2 ppm NO_2_ was significantly higher at 5.6, demonstrating minimal cross-sensitivity to other gases. This remarkable selectivity, combined with the enhanced sensitivity and improved response kinetics, underscores the effectiveness of Pd nanoparticle decoration in optimizing the NO_2_ sensing properties of V_2_O_5_/PSi composites.

The long-term stability of the Pd-V_2_O_5_/PSi composite, as shown in Figure 7d, was evaluated by exposing it to 2 ppm NO_2_ over a 30-day period. The sensor exhibited highly consistent performance throughout the testing duration, with a maximum response of 5.77, a minimum response of 5.39, and an average response of 5.6. The response fluctuation remained within a narrow range of ±3.75%, demonstrating excellent stability and reliability. The stability is further enhanced by the robust structure of the PSi substrate and the strong interfacial interactions between Pd, V_2_O_5_, and PSi, which prevent degradation of the sensing material over time.

### 3.4. Mechanism of Gas-Sensing

V_2_O_5_, as a typical n-type MOS, initially follows the well-established depletion model for its NO_2_ sensing mechanism. However, the introduction of Pd decoration and the formation of the V_2_O_5_/PSi heterojunction induce a transition to p-type behavior under NO_2_ exposure, as confirmed by resistance dynamics. When exposed to ambient air, oxygen molecules chemisorb onto the surface of V_2_O_5_ nanorobs and are subsequently transformed into reactive oxygen species (O_2_^−^, O^−^, and O^2−^) by capturing electrons from the conduction band of V_2_O_5_. This process forms an electron depletion layer near the surface of the V_2_O_5_ nanorobs, as described by the following reactions [32]:

O_2(gas)_ → O_2(ads)_, (1)

O_2(gas)_ + e^−^ → O_2_^−^_(ads)_, (2)

O_2_^−^_(ads)_ + e^−^ → 2O^−^_(ads)_, (3)

O^−^_(ads)_ + e^−^ → O^2−^_(ads)_, (4)

The NO_2_-sensing mechanism of the Pd-V_2_O_5_/PSi composite is governed by hole-dominated conduction, as evidenced by the observed resistance decrease under NO_2_ exposure. When exposed to NO_2_, the molecules preferentially adsorb onto the V_2_O_5_/PSi surface due to their significantly higher electron affinity compared to oxygen molecules [30]. This preferential adsorption occurs under dynamic adsorption–desorption equilibrium conditions [31,32,33], where NO_2_ molecules effectively compete with oxygen for surface active sites. The adsorption process extracts electrons from the valence band of V_2_O_5_, resulting in two significant phenomena: the expansion of the hole accumulation layer and the formation of an inversion layer where holes become the dominant charge carriers. Additionally, NO_2_ molecules interact with pre-chemisorbed oxygen ions, facilitating further electron trapping, as described by the following reactions:

NO_2(gas)_ + e^−^ → NO_2_^−^_(ads)_ + h^+^, (5)

NO_2(ads)_ + O_2_^−^_(ads)_ + e^−^ → NO_2_^−^_(ads)_ + 2O^−^_(ads)_ + h^+^, (6)

NO_2(ads)_ + O^−^_(ads)_ + e^−^ → NO_2_^−^_(ads)_ + O^2−^_(ads)_ + h^+^. (7)

The presence of a hole accumulation layer in the Pd-V_2_O_5_/PSi heterostructure enhances its capability to adsorb oxidizing gases, particularly NO_2_. This enhancement is attributed to the increased number of adsorption sites created by the heterojunctions, which significantly contributes to the improved sensor performance. From the perspective of MOS gas sensor operation principles, specific surface area plays a crucial role in determining gas sensing performance. The V_2_O_5_/PSi heterostructure exhibits a high specific surface area, providing abundant active sites for gas molecule adsorption. This structural characteristic enables increased NO_2_ molecule adsorption on the material surface, enhanced electron transfer, and further widening of the depletion region. These combined effects result in a higher sensitivity and response to NO_2_ detection.

**Figure 8:**
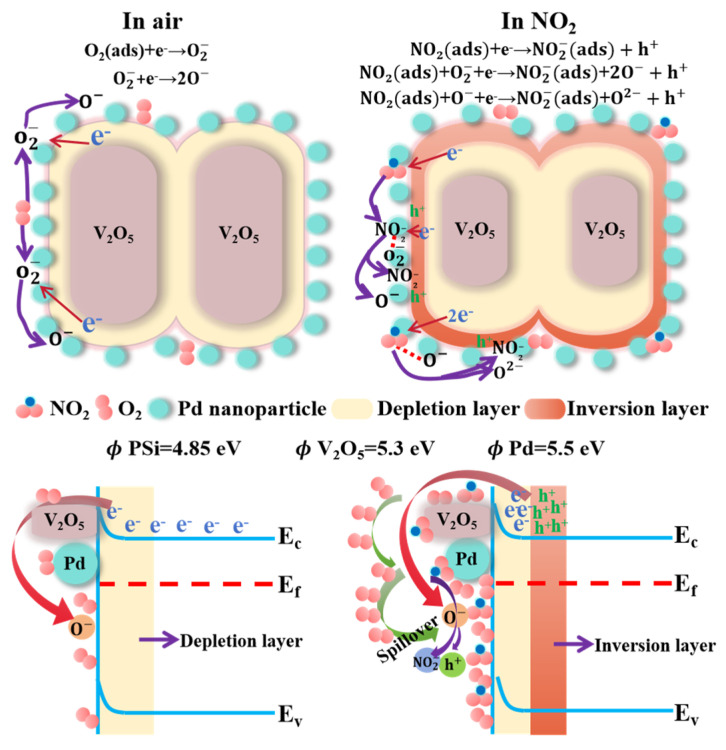
Schematic illustration of the gas-sensing mechanism of Pd-V_2_O_5_/PSi in air and NO_2_.

Based on the sensing mechanism of Pd-V_2_O_5_/PSi, the improved sensing characteristics can be attributed to three primary mechanisms, as illustrated in Figure 8, which depicts the gas-sensing mechanism of Pd-V_2_O_5_/PSi in air and NO_2_ environments. Firstly, the PSi substrate plays a crucial role in the NO_2_ sensing process. The high specific surface area and excellent adsorption properties of PSi provide abundant diffusion pathways and adsorption sites for NO_2_ gas molecules. This porous architecture facilitates both the penetration of NO_2_ molecules into the composite material and their subsequent interaction with V_2_O_5_. Moreover, the porous structure enables rapid diffusion and desorption of gas molecules, thereby enhancing the response speed and recovery capability. Secondly, the formation of a Schottky junction at the Pd-V_2_O_5_ interface significantly contributes to the enhanced sensing performance. The work function difference between Pd (~5.5 eV) and n-V_2_O_5_ (~5.3 eV) induces electron transfer from V_2_O_5_ to Pd until Fermi level alignment is achieved. This process creates a hole accumulation layer at the Pd-V_2_O_5_ interface, which stabilizes the inversion layer and enhances hole conduction under NO_2_ exposure. The metal spillover effect generates additional active adsorption sites on Pd nanoparticles, facilitating enhanced gas molecule adsorption [20]. The increased concentration of adsorbed species enables NO_2_ molecules to not only directly capture electrons from the V_2_O_5_ conduction band but also react with the pre-adsorbed oxygen ions, leading to enhanced sensing response, as evidenced by the results shown in Figure 7a. Thirdly, the catalytic properties of Pd nanoparticles play a significant role in improving sensor performance. Pd nanoparticles reduce the activation energy for chemical adsorption of gas molecules and accelerate the rate of chemical reactions. This catalytic effect lowers the optimal operating temperature of the Pd-V_2_O_5_/PSi while simultaneously reducing response and recovery times. The enhanced coverage of oxygen ions on the sensor surface contributes to the improved NO_2_ sensing capability. Furthermore, Pd nanoparticles facilitate the adsorption/desorption of NO_2_, molecule–ion conversion of oxygen, and the interaction between NO_2_ and chemisorbed oxygen ions, resulting in significantly improved response and recovery characteristics. The synergistic combination of these mechanisms enables the Pd-V_2_O_5_/PSi to achieve superior response characteristics and shorter response/recovery times at room temperature compared to the undecorated V_2_O_5_/PSi.

### 3.5. Role of Pd Decoration in Enhancing Sensing Performance

**Figure 9:**
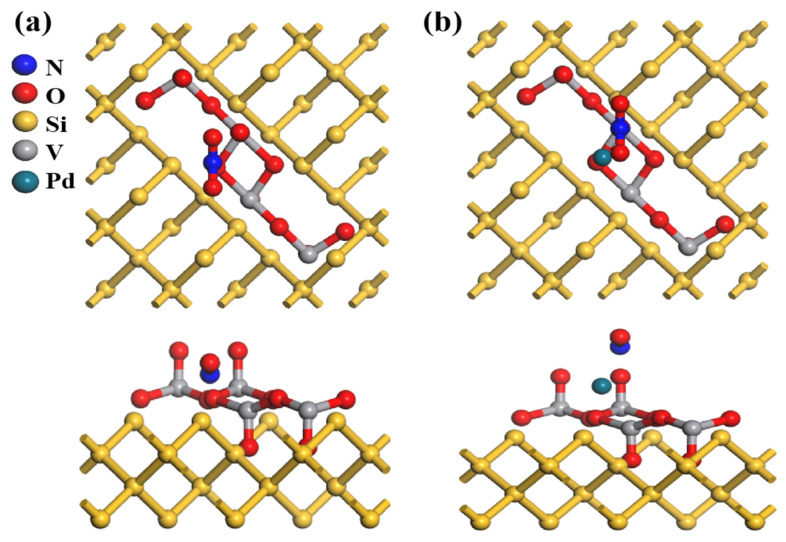
Atomic models (top and side views) of (**a**) V_2_O_5_/PSi and (**b**) Pd-V_2_O_5_/PSi in an NO_2_ atmosphere.

In the atomic models shown in Figure 9a,b, the undecorated V_2_O_5_/PSi system in Figure 9a shows NO_2_ adsorption primarily on the oxygen atoms of the V_2_O_5_ framework, forming stable but weak bonds with minimal structural changes. In contrast, the Pd-V_2_O_5_/PSi system in Figure 9b exhibits stronger NO_2_–surface interactions, with Pd atoms serving as additional adsorption sites that facilitate electron transfer and enhance adsorption capacity. This modification strengthens NO_2_ adsorption by altering the local bonding environment and increasing surface reactivity, suggesting improved electronic and catalytic performance.

**Figure 10:**
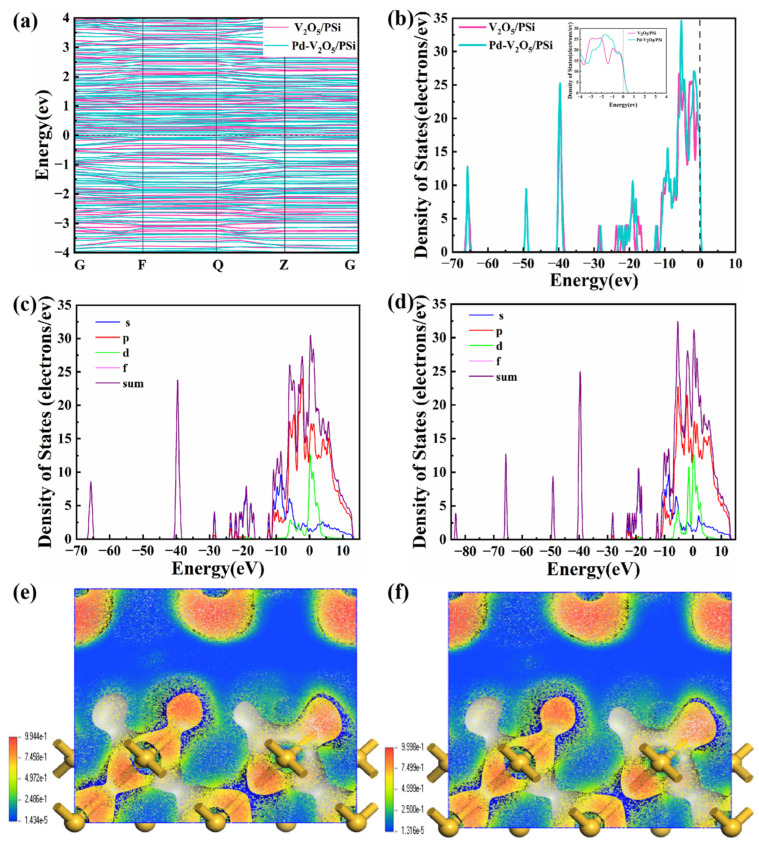
Electronic interactions under NO_2_ adsorption: (**a**) Energy band structure and (**b**) DOS of V_2_O_5_/PSi and Pd-V_2_O_5_/PSi; PDOS and ELF plots of (**c**,**e**) V_2_O_5_/PSi and (**d**,**f**) Pd-V_2_O_5_/PSi.

The experimental results (Figure 7) demonstrate that the decoration of Pd nanoparticles significantly enhances the gas sensing performance of the V_2_O_5_/PSi composite. As shown in the dynamic response curve of Pd-V_2_O_5_/PSi to 2 ppm NO_2_ at 25 °C (Figure 7b), the resistance decreases upon exposure to NO_2_, and after the response and recovery process, the resistance returns to its initial value. This behavior confirms the reversible nature of the sensing mechanism and the stability of the Pd-V_2_O_5_/PSi composite. Figure 10a–f presents a comprehensive analysis of the structural, electronic, and adsorption properties of V_2_O_5_/PSi and Pd-V_2_O_5_/PSi composites in an NO_2_ environment, further elucidating these effects.

In Figure 10a, the NO_2_ adsorption of the V_2_O_5_/PSi system induces minimal changes, reflecting weak adsorption and limited electronic coupling. In contrast, the Pd-V_2_O_5_/PSi system exhibits a reduced bandgap and localized electronic states near the Fermi level, enhancing conductivity. Pd decoration introduces additional energy levels near the CB and increases DOS peaks (Figure 10b), indicating stronger electronic coupling and enhanced electron density. These changes improve NO_2_ adsorption and reactivity, highlighting Pd’s role as an electronic coupling bridge.

The PDOS analysis of NO_2_-adsorbed systems reveals critical electronic modifications induced by Pd decoration. In the PSi-V_2_O_5_/NO_2_ system (Figure 10c), the s-orbital dominates at mid-to-low energies (−30 to +15 eV), reflecting Si–O–V_2_O_5_ interfacial interactions. The p-orbital hybridizes strongly near the Fermi level (0 eV) and spans −10 to +5 eV, facilitating charge transfer. The d-orbital contributes broadly (−20 to +10 eV), primarily from V_2_O_5_’s transition metal centers. Upon Pd decoration (Figure 10d), the p-orbital exhibits intensified hybridization near the Fermi level, amplifying oxygen-mediated charge delocalization. Most critically, the d-orbital develops a prominent peak at 0 eV, attributed to Pd 4d states, which directly bridges the CB and NO_2_ antibonding orbitals. The enhanced Pd 4d contribution correlates with the experimentally observed 3.1-fold improvement in sensor response (Section 3.3), demonstrating that Pd decoration facilitates interfacial charge dynamics rather than altering the bulk band structure. These findings are consistent with the improved sensitivity and selectivity observed in Section 3.3, where Pd-V_2_O_5_/PSi exhibited faster response and recovery times compared to the undecorated V_2_O_5_/PSi composite.

The ELF shown in Figure 10e,f provides further insights. In Figure 10e, the V_2_O_5_/PSi system shows localized electron density primarily in specific regions of the material surface, where yellow and red regions represent higher electron density, while blue areas indicate lower electron density. The color distribution highlights the regions where the composite surface interacts most strongly with NO_2_ molecules. The range of electron density values, from 1.434 × 10^−5^ to 9.944 × 10^−1^, further emphasizes localized electronic behavior near the surface, suggesting that the V_2_O_5_/PSi composite exhibits strong surface interactions with the gas molecules. In contrast, the Pd-V_2_O_5_/PSi system in Figure 10f exhibits a more extensive electron density in the yellow and red regions, indicating an increase in overall electron localization. The range of electron density values from 1.316 × 10^−5^ to 9.998 × 10^−1^ highlights a more concentrated electron distribution, especially at the Pd sites and the adjacent material surface. This enhanced electron localization suggests that Pd decoration not only increases the material’s interaction with gas molecules but also improves its electron transfer capability, as evidenced by the improved sensing performance in Section 3.3. As a result, the Pd-V_2_O_5_/PSi composite demonstrates stronger interactions between Pd and NO_2_, significantly improving the material’s electronic properties, sensitivity, and selectivity to NO_2_.

Collectively, these results reveal that Pd decoration markedly enhances the electronic structure, adsorption capacity, and reactivity of the V_2_O_5_/PSi composite. The introduction of localized states, reduced bandgap, increased DOS peaks, and enhanced electron localization facilitate efficient electron transfer and stabilize NO_2_ interactions. These findings are consistent with the experimental results in Section 3.3, where Pd-V_2_O_5_/PSi exhibited superior sensing performance, including faster response/recovery times and higher sensitivity to NO_2_, underscoring its potential for advanced gas sensing and catalytic applications by leveraging its improved conductivity and reactivity.

## 4. Conclusions

In this study, a Pd-V_2_O_5_/PSi composite was successfully synthesized via magnetron sputtering, and its application as a high-performance NO_2_ gas sensor at room temperature was systematically investigated through a combination of experimental characterization and first-principles calculations. The experimental results demonstrated that the decoration of Pd nanoparticles significantly enhanced the sensitivity, selectivity, and response/recovery characteristics of the composite material. Specifically, the Pd-V_2_O_5_/PSi composite exhibited a 3.1-fold increase in response to 2 ppm NO_2_ at 25 °C compared to the undecorated V_2_O_5_/PSi, along with excellent reproducibility and long-term stability. First-principles calculations provided a detailed understanding of the structural, electronic, and adsorption properties of the Pd-V_2_O_5_/PSi system in NO_2_ environments. The results revealed that Pd decoration introduces localized electronic states near the Fermi level, reduces the bandgap, and enhances electron density, thereby improving the material’s conductivity and adsorption capacity. ELF analysis further confirmed that Pd strengthens the interaction between NO_2_ and the composite surface, highlighting its role as an electronic coupling bridge. These theoretical insights, combined with experimental findings, elucidate the mechanisms underlying the enhanced gas sensing performance of the Pd-V_2_O_5_/PSi composite. This research not only advances the development of high-performance NO_2_ gas sensors but also provides fundamental insights into the design and optimization of metal oxide-based sensing materials. The synergistic combination of experimental and theoretical approaches offers a robust framework for understanding the underlying mechanisms of gas adsorption and sensing mechanisms at the atomic level.

## Figures and Tables

**Table 1:** Comparison of NO_2_ gas-sensing performances of Pd-V_2_O_5_/PSi with those reported in the previous literature.

Materials	Working Temp. (°C)	Response	NO_2_ Conc. (ppm)	Ref.
ZnO/SnO_2_	90	25	0.5	[7]
TiO_2_ NTs/rGO	Room temperature	19.1	1	[13]
Au@WO_3_	100	2	0.25	[30]
Graphene/ZnO	300	9.5	50	[31]
Pd-V_2_O_5_/PSi	25	5.6	0.25	This work

## Data Availability

The data presented in this study are available on request from the corresponding author.
